# Primary basal cell carcinoma of the caruncle: case report and review of the literature

**DOI:** 10.5935/0004-2749.2022-0357

**Published:** 2024-03-05

**Authors:** Elizabeth Cecilia Roque-Choque, Jorge Ramiro Villalobos-Espinoza, Isabel Silva-Ocas, Rosa Alvarado-Villacorta, Pedro Muro-Mansilla

**Affiliations:** 1 Instituto Nacional de Oftalmología “Dr. Francisco Contreras Campos”, Lima, Perú; 2 Universidad Peruana Cayetano Heredia, Lima, Perú; 3 Departamento de Patología Ocular “Dr. José Antonio Avendaño Valdez”, Instituto Nacional de Oftalmología “Dr. Francisco Contreras Campos”, Lima, Perú; 4 Dirección ejecutiva de Investigación y docencia especializada en oftalmología y desarrollo de Tecnologías, Instituto Nacional de Oftalmología “Dr Francisco Contreras Campos”, Lima, Perú; 5 Unidad de Investigación Clínica, Scientia Clinical and Epidemiological Research Institute, Trujillo, Perú; 6 Servicio de Córnea y Cirugía Refractiva, Asociación para Evitar la Ceguera en México I.A.P. Ciudad de México, México; 7 Universidad Nacional Autónoma de México, Ciudad de México, México; 8 Departamento de Atención Especializada en Oculoplástica y Oncología Ocular, Instituto Nacional de Oftalmología “Dr. Francisco Contreras Campos”, Lima, Perú

**Keywords:** Conjunctival diseases, Eye neoplasms, Sebaceous gland neoplasms, Conjunctival neoplasms, Carcinoma, basal cell, Diagnosis, differential, Humans, Case reports

## Abstract

We present a rare case of primary caruncle basal cell carcinoma (BCC), a condition with limited occurrences. Our patient, an 80-year-old woman without prior ocular pathological history, presented a 2x2mm pedunculated blackish nodular lesion on the caruncle of her left eye, without local conjunctival or cutaneous involvement. Histological analysis following complete excision confirmed the presence of basal cell carcinoma within the caruncle. Over a span of 30 months, no recurrence has been observed. While scant cases are documented in the literature, we conducted a review of these instances. Despite its infrequent manifestation, this condition should be taken into account when evaluating caruncular tumors, given its tendency to invade the orbit. Complete excision with free surgical margins is the treatment of choice, and adjuvant radiotherapy or chemotherapy might be considered.

## INTRODUCTION

The caruncle, positioned at the inner edge of the eye, is a pink, ovoid conjunctival relief, measuring approximately 5 mm in height and 3 mm in width. It serves to retain tears and secrete mucus. Like the skin, it contains hair, sebaceous and sweat glands, and accessory lacrimal glands. Tumor formations originating here can encompass a diverse array of lesions, resembling those found in the skin, conjunctiva, or lacrimal gland^(1,2)^.

Basal cell carcinoma (BCC) constitutes nearly 80% of non-melanoma skin cancers^(3)^. However, primary caruncle BCCs are extremely rare, mostly resulting from the local spread of adjacent skin neoplasms^(1)^, accounting for a round 4% of excised conjunctival lesions^(4)^. While its metastatic risk is low, making its mortality rate minimal, it still poses a significant local threat due to its invasive nature^(5)^.

In this report, we present a patient’s case involving primary caruncle BCC, along with a comprehensive literature review.

## CASE REPORT

An 80-year-old woman from Ancash-Peru, who is undergoing treatment for arterial hypertension, has no previous history of ocular issues, cutaneous conditions, or visceral malignancies. She sought medical attention due to a progressively growing blackish lesion on the conjunctiva near the inner canthus of her left eye (OS). The lesion had been developing for 2 months and was associated with itching and the discharge of whitish mucous.

**Figure 1:**
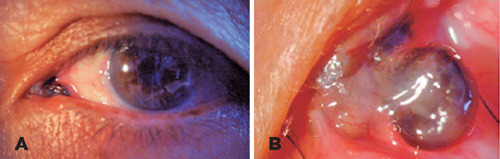
A) Pigmented lesion with progressive growth in the caruncle and conjunctiva area near the inner canthus of the left eye. B) Blackish caruncle with telangiectatic vessels, with a protruding blackish and pedunculated nodular lesion

**Figure 2:**
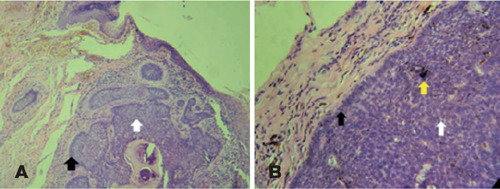
A) Nests of basaloid cells, hyperchromatic nuclei (white arrow), and peripheral palisade (black arrow) (HE, 100X). B) Hyperchromatic nuclei, scarce and poorly defned cytoplasm, absence of intercellular bridges (white arrow), and peripheral palisade (black arrow), characteristic of basal cell carcinoma. Presence of melanic pigment is observed within the tumor (yellow arrow) and adjacent stromal tissue (HE, 400X)

**Figure 3:**
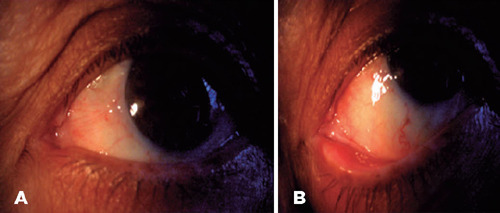
At 30 months of follow-up. A) Clinical photo of the left eye without evidence of recurrence. B) At higher magnifcation, the inner canthus without abnormal vessels or pigmentation, or new tissue growth

Biomicroscopy revealed a blackish lesion in the caruncular region with telangiectatic vessels, from which a pedunculated nodular lesion of approximately 2x2mm protruded, with erosion on its outer surface. Importantly, there was no adherence to deeper planes or interference with ocular movements. No other abnormalities were detected in either the anterior or posterior segment (Figure 1). To address this, an excisional biopsy was conducted with wide margins. This procedure yielded two specimens: the first was a 9x6.5x4.5mm blackish irregularly shaped and surfaced fragment with a firm consistency, while the second was a 1x0.5x0.5mm conjunctival fragment from the margin with similar characteristics. Both specimens were forwarded to the Ocular Pathology Service “Dr. José Antonio Avendaño Valdez” of the National Institute of Ophthalmology. Histopathological analysis confirmed the first specimen to be pigmented BCC, while the second specimen indicated an absence of neoplastic infiltration (Figure 2). During the 30-month follow-up period, no recurrences were reported (Figure 3).

## DISCUSSION

The caruncle serves as a transition zone between the skin and the conjunctiva, causing the spectrum of lesions to arise there to be highly varied, thereby complicating clinical diagnoses^(1)^. Only 50% of preoperative clinical diagnoses align with histopathological findings, while 29% of malignant lesions are clinically misdiagnosed as benign^(2)^. Within a 24-year case series of caruncle lesions, 96% of the lesions were benign, 1.7% premalignant, and 2.4% malignant. Among the malignancies, BCC constituted 0.7% of this group^(2)^.

BCC commonly develops in areas exposed to sunlight, primarily affecting fair-skinned elderly individuals. Notably, the periorbital region, lower eyelid, and medial canthus tend to be the areas most impacted^(3,5)^. Despite its limited metastasis capacity, BCC ranks as the third most prevalent invasive malignant neoplasm affecting the orbit (10%)^(6)^. Its potential to extend intracranially poses a serious threat^(3)^, largely attributed to its perineural and perivascular growth pattern^(7)^.

Microscopically, BCC is composed of cells with large, elongated nuclei featuring prominent palisading. The cytoplasm can appear inconspicuous, pale, or lightly eosinophilic, while instances of mitoses and single-cell apoptosis are usually observed, sometimes prominently. BCC can manifest various growth patterns, encompassing both nonaggressive and aggressive types. Notably, BCC exhibits cytokeratin profiles characterized by the expression of CK5/6 and CK14, as well as CK20- and Ber-EP4+. Multiple dermatological conditions share histological similarities with BCC, potentially leading to misdiagnoses and consequent unnecessary excisions or delayed assessment of metastatic disease. Differentiating between these entities requires a combination of clinical correlation, identification of histologic features, and, in difficult cases, utilization of immunohistochemistry. An example includes trichoblastoma, a benign lesion characterized by the presence of mesenchymal bodies, a reaction involving giant cells, mitotically inactivity, and absence of tumor necrosis or cytological atypia, along with PHLDA1+, CK20+, AR-. Another is sebaceous carcinoma, a malignant lesion displaying higher cytologic atypia and reduced basaloid morphology in comparison to BCC. Sebaceous carcinoma exhibits diffuse AR+ (versus focally positive in BCC) and is characterized by low molecular-weight CK+, EMA+, and Ber-EP4^(8)^.

Primary treatment relies on complete surgical excision, although challenges may arise in obtaining free surgical margins^(5)^, often due to inadvertent damage to the canalicular system^(1)^. In specific instances, Mohs micrographic surgery offers an alternative, as it preser-

ves greater amounts of normal tissue while ensuring adequate removal^(5)^. To curb the risk of recurrence or orbital infiltration, supplementary approaches encompass radiation therapy and intra-arterial infusion of antineoplastic agents into the tumor site^(9)^. In our patient’s case, a complete excision with margins free of lesion was performed. Throughout a 30-month follow-up, no signs of recurrence surfaced, rendering irradiation unnecessary. Remarkably, our patient exhibited no signs of skin involvement or orbital invasion. Consequently, this instance appears to be the first case of primary BCC of the caruncle within the Latin American patient population.

A search was conducted across MedLine/PubMed, Scopus, and the Virtual Health Library databases. This endeavor yielded 16 publications, encompassing a total of 24 cases dating back to 1977. Out of these, 11 publications provided comprehensive insights into the characteristics of BCC and its treatment strategies (Table 1).

**Table 1:** Summary of reported cases of primary basal cell carcinoma (BCC) of the caruncle

Author	Age (years) /Sex	Country	Clinical appearance	Type of BCC	Orbital invasion	Treatment	Recurrence (tracing)
Poon et al., 1997^(10)^	74 M	Australia	TI = 6 m LE, multilobed, vascularized, pink nodule	Solid - microcystic	No	Excision	Not stated
Meier et al., 1998^(11)^	24 M	Germany	TI = 3 m LE, nodule, vascularized, white with red center	Solid-cystic	No	Excision	No (14 m)
Mencía-Gutiérrez et al., 2005^(12)^	80 M	Spain	TI = 5 m LE, irregular appearance, blackish brown, vascularized	Nodular	No	Excision	No (7 y)
Østergaard et al., 2005^(2)^	60 F	Denmark	TI = weeks. LE, lobulated, cystic nodule, vascularized, pale	Not described (Infiltrative islands of basaloid tumor cells)	Ye s	Excision	Yes (5,5 y)
Rossman et al., 2006^(13)^	82 M	USA	TI = 2 – 3 m LE, solid, pale nodule	Nodular	No	Excision, adjuvant radiotherapy.	No (6 m)
Kaeser et al., 2006^(14)^	72 F	Switzerland	Cystic nodule	Not described	Not described	Excision	No
Kaeser et al., 2006^(14)^	52 M	Switzerland	Blackish cystic nodule	Pigmented macronodular	Not described	Excision	No
Lee et al., 2010^(9)^	73 M	China	LE, Lobulated, blackish brown	Pigmented	No	Excision, Intraarterial chemotherapy	Yes (22 m)
Fino et al., 2013^(15)^	24 F	Italy	TI = 6 m RE, lobulated, brown	Solid, pigmented	No	Excision	No (12 m)
Ugurlu et al., 2014^(16)^	67 M	Turkey	TI = 12 m RE, Vascularized, pink	Nodular	Ye s	Excision, Radiotherapy refused	No (33 m)
Mihailovic et al., 2019^(17)^	58 M	Germany	LE, brown, impregnated with telangiectatic vessels	Solid - Nodular	No	Excision	No (6 m)
Present Case	80 F	Peru	TI = 2 m LE, rounded blackish	Pigmented	No	Excision	No (30 m)

TI= time of illness; LE= left eye; RE= right eye; m= months; y= years.

Upon our examination, a male predominance was evident, with a ratio of 3:1 compared to women. The age range spanned from 24 to 82 years, with an average age of 60.5 years. The left eye (OS) exhibited higher frequency, and the mean duration of manifestation was approximately 5 months. The clinical presentation displayed a diverse range of colors and shapes. Among the patients, two (16%) had orbital invasion at the time of initial presentation^(6,16)^. To elaborate, the first patient, as described by Østergaard^(6)^, developed a second BCC in the lower fornix (3 years later), along with recurrence at the original site (5.5 years afterward). In the case of the second patient, reported by Lee^(9)^, a recurrent BCC emerged 3 months after undergoing chemotherapy. Subsequent resection of the lesion led to a recurrence-free period lasting 22 months.

In all cases, lesion excision was performed. Rossman recommended radiotherapy due to uncertainties surrounding lesion margins, and they reported no recurrences within a 6-month follow-up period. Ugurlu^(16)^ suggested adjuvant radiotherapy post-surgery, citing orbital invasion and evident infiltration of the medial rectus muscle. However, the patient declined this option, and over the course of 33 months, no recurrence transpired.

Despite its rarity, primary caruncular BCC should be considered in the context of differential diagnosis due to its potential orbital invasion. The treatment of choice involves complete excision with tumor-free surgical margins. Should this prove unattainable, the consideration of radiotherapy or adjuvant chemotherapy is warranted. Cases of recurrence have been documented, underscoring the imperative for long-term follow-up.
